# Neoadjuvant therapy with cadonilimab in a patient with MSI-H/dMMR colorectal cancer: a case report

**DOI:** 10.3389/fonc.2025.1693932

**Published:** 2026-01-05

**Authors:** Jingrui Zhou, Weimin Chen, Jue Wang, Ziwei Chen, Guanghui Guo, Tao Yang, Tao Shen

**Affiliations:** Department of Colorectal Surgery, Yunnan Cancer Hospital, The Third Affiliated Hospital of Kunming Medical University, Peking University Cancer Yunnan Hospital, Kunming, Yunnan, China

**Keywords:** colorectal cancer, cadonilimab, immune checkpoint inhibitors, neoadjuvant immunotherapy, pathological complete response, programmed death-1 (PD-1), cytotoxic T-lymphocyte-associated antigen 4 (CTLA-4)

## Abstract

Neoadjuvant immunotherapy for mismatch repair-deficient/microsatellite instability-high (dMMR/MSI-H) colorectal cancer continues to accumulate compelling clinical evidence. This report presents the case of a 37-year-old female who presented with moderate anemia (hemoglobin: 69 g/L) and was subsequently diagnosed with dMMR/MSI-H descending colon cancer. Following nine cycles of cadonilimab, a novel PD-1/CTLA-4 bispecific antibody, the patient achieved significant tumor regression and subsequent pathological complete response (pCR) after surgery, with a favorable safety profile observed throughout the treatment course. This case provides valuable clinical evidence supporting the efficacy and safety of cadonilimab in the neoadjuvant setting for dMMR/MSI-H colorectal cancer.

## 1 Background

Colorectal cancer (CRC) holds a prominent position among gastrointestinal malignancies. According to the latest 2022 report by the International Agency for Research on Cancer (IARC) of the World Health Organization (WHO), it was estimated that there would be 20 million new cancer cases and 9.7 million cancer-related deaths globally. Among these, CRC was one of the top three cancer types in 2022 (1). CRC patients with mismatch repair deficiency (dMMR)/microsatellite instability-high (MSI-H) are associated with high tumor mutational burden (TMB) and increased immunogenicity, which represent only 5-15% of all CRC cases (2). Locally advanced CRC (LACRC) patients with dMMR/MSI-H typically exhibit poor sensitivity to conventional neoadjuvant chemoradiotherapy (nCRT) or neoadjuvant chemotherapy (nCT) (3). Fortunately, the advent of neoadjuvant immunotherapy (nIT) has provided new hope for these patients (4). Recently, studies on nIT for advanced dMMR/MSI-H CRC, including the NICHE trial(ClinicalTraial.gov NCT03026140;registered on January 23,2017.), the NICHE-2 trial the NICHE-2 trial (ClinicalTrials.gov NCT03026140; registered on January 23, 2017.), the PD-1 blockade trial in dMMR rectal cancer (ClinicalTrials.gov NCT03026140; registered on November 18, 2019.) and the PICC trial(ClinicalTrials.gov NCT05962200;registered on July 25, 2023.) (5–7). Notably, clinical findings from the NICHE study suggest that nIT has demonstrated promising clinical efficacy and surgical outcomes in dMMR CRC patients, with a 100% pathological response rate and nearly 60% pathological complete response (pCR) rate (8). Immune checkpoint inhibitors (ICIs), such as antibodies targeting PD-1, PD-L1, or CTLA-4, have become effective treatment options for a variety of cancers (9). In the review published by our center in 2022, five retrospective studies conducted in the China, the United States, and Belgium during 2019 and 2020 were listed. A total of 12 subjects with MSI-H/dMMR LACRC were treated with nIT, reporting a clinical complete response (CR) rate of over 80% (3). Additionally, a recent case report from our center documented a locally advanced ascending colon cancer patient with MSI-H/dMMR achieved pCR after neoadjuvant Envafolimab monotherapy (10). These findings underscore the significant benefit of nIT for such patients. Recently, clinical guidelines have been continually updated. In the Chinese Society of Clinical Oncology (CSCO) Guidelines for Diagnosis and Treatment of Colorectal Cancer (2021), it is suggested to use the PD-1 inhibitors for MSI-H/MMR patients undergoing conversion therapy or palliative care. The CSCO Guidelines for Diagnosis and Treatment of Colorectal Cancer (2023,2024 and 2025) mentions that in the treatment principles for patients with cT_1-2_N rectal cancer, ICI treatment may be considered for MMR/MSL-H patients, followed by an assessment to determine the necessity of surgery and the formulation of a surgical plan, based on the results from clinical studies on the treatment of locally advanced rectal cancer (LARC) conducted by the MSKCC and Sun Yat-sen University Cancer Center and after the discussion by MDT. It is evident that immunotherapy holds a significant position in the treatment of CRC patients with MSI-H/MMR.

Cadonilimab is a PD-1/CTLA-4 bispecific antibody that works by simultaneously targeting both PD-1 and CTLA-4 immune checkpoints. This dual-targeting approach reduces the activation of immune suppressive pathways in tumor cells, while enhancing T cell-mediated immune responses. This “dual checkpoint blockade” mechanism indirectly releases and activates immune cells, thereby boosting immune activity and strengthening the anti-tumor effect (11). The unique design of Cadonilimab lies in its tetravalent structure, which enables multivalent binding to T cells co-expressing PD-1 and CTLA-4 within the tumor micro-environment. This design not only retains the efficacy of combined treatment with PD-1 and CTLA-4 inhibitors but also optimizes the Fc region to eliminate antibody-dependent cellular cytotoxicity (ADCC) and antibody-dependent cellular phagocytosis (ADCP) effects, which reduce immune-related adverse reactions and improving stability and anti-tumor activity. In the context of dMMR/MSI-H CRC, whether Cadonilimab as a nIT can achieve comparable or superior surgical outcomes and safety profiles still need further exploration and research. This report presents a case with MSI-H/dMMR CRC, who achieved pCR after receiving neoadjuvant Cadonilimab as monotherapy. This case serves as a reference for future studies.

## 2 Case study

The patient, a 37-year-old woman, presented with sallow skins on her face and hands, which was first noticed by her family and friends in February 2024. She sought medical evaluation and took blood routine test at a local hospital, with a testing result of HB level at 69 g/L. Then, she underwent a colonoscopy at the First Affiliated Hospital of Kunming Medical University, where a mass was identified in the descending colon. Case examination confirmed the diagnosis of adenocarcinoma. Subsequently, the patient was referred to our hospital for further management.

## 3 Physical examination

Physical examination: Mild tenderness was presented in the left lower abdomen without rebound tenderness. No obvious abnormalities were noted on palpation of the left lower abdomen and the rectal exam. Medical history: The patient has a history of two cesarean sections, appendectomy, and cholecystectomy. Personal history: No smoking and alcohol history. Physiological condition: Body Mass Index (BMI): 28.30 kg/m²; weight: 68 kg; height: 155 cm; BAS: 1.7845 m²; ECOG-PS: 1; NRS 2002: 1; ADL: 0. In short, the preliminary diagnosis was “descending colon malignant tumor”.

## 4 Diagnosis & treatment

**Figure 1:**
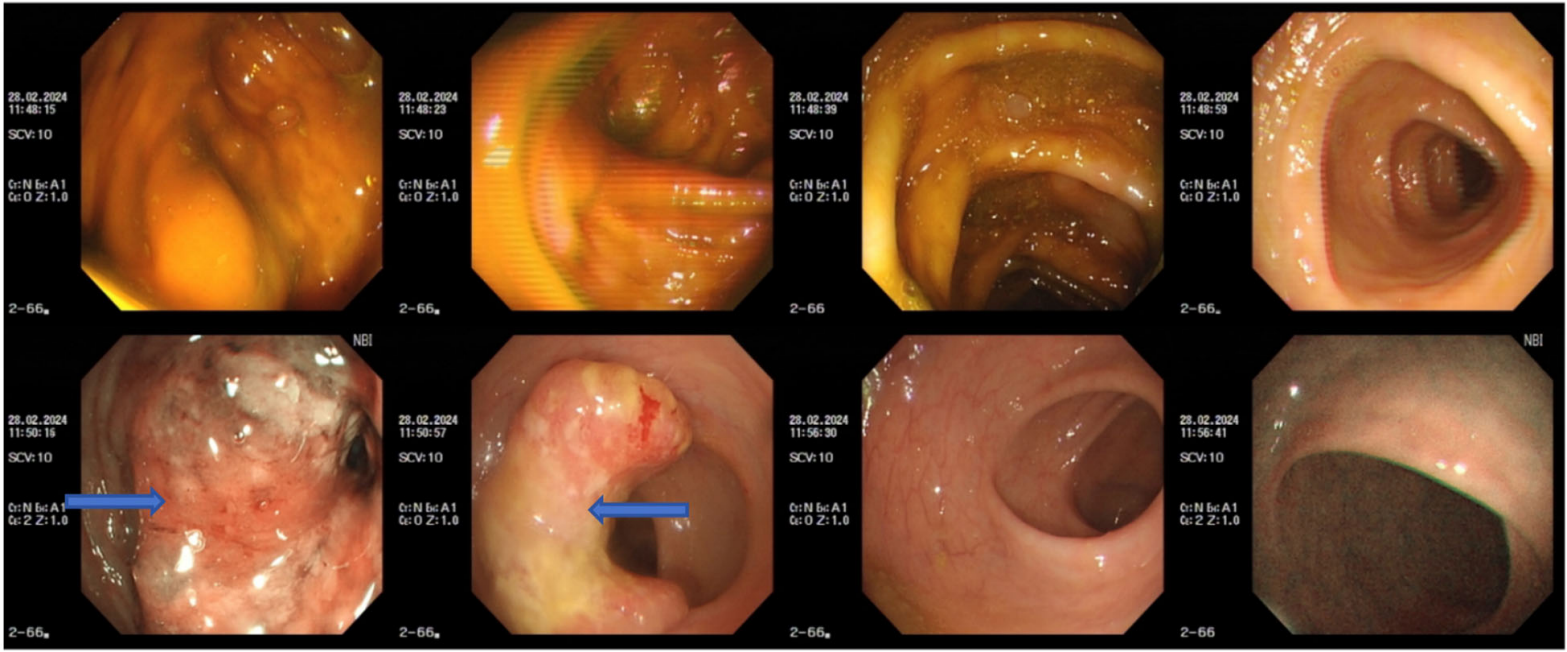
Endoscopic image from Feb 28, 2024. Colonoscopy showing the descending colon tumor from different angles.

First colonoscopy on Feb, 2024: A mass approximately 3×4 cm in size was observed in the descending colon at 30 cm from the anus. The mass appeared edematous and congested, with a central ulcer covered with a dirty coating, invading about half of the bowel circumference. See **Figure 1**.

**Figure 2:**
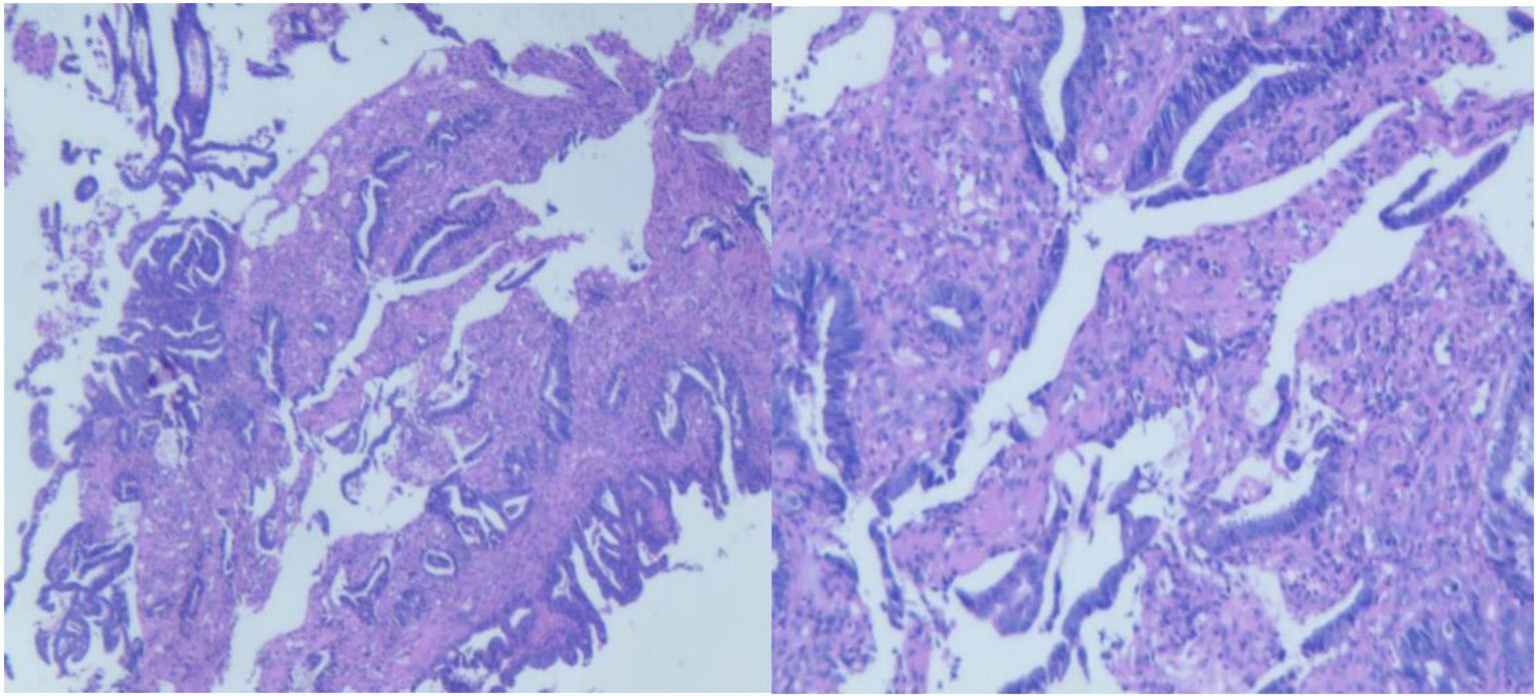
(Time) Pathological image of the descending colon tumor from the initial diagnosis.

Case examination in Feb, 2024: Adenocarcinoma in the descending colon. See **Figure 2**. Immunohistochemistry: dMMR.

**Figure 3:**
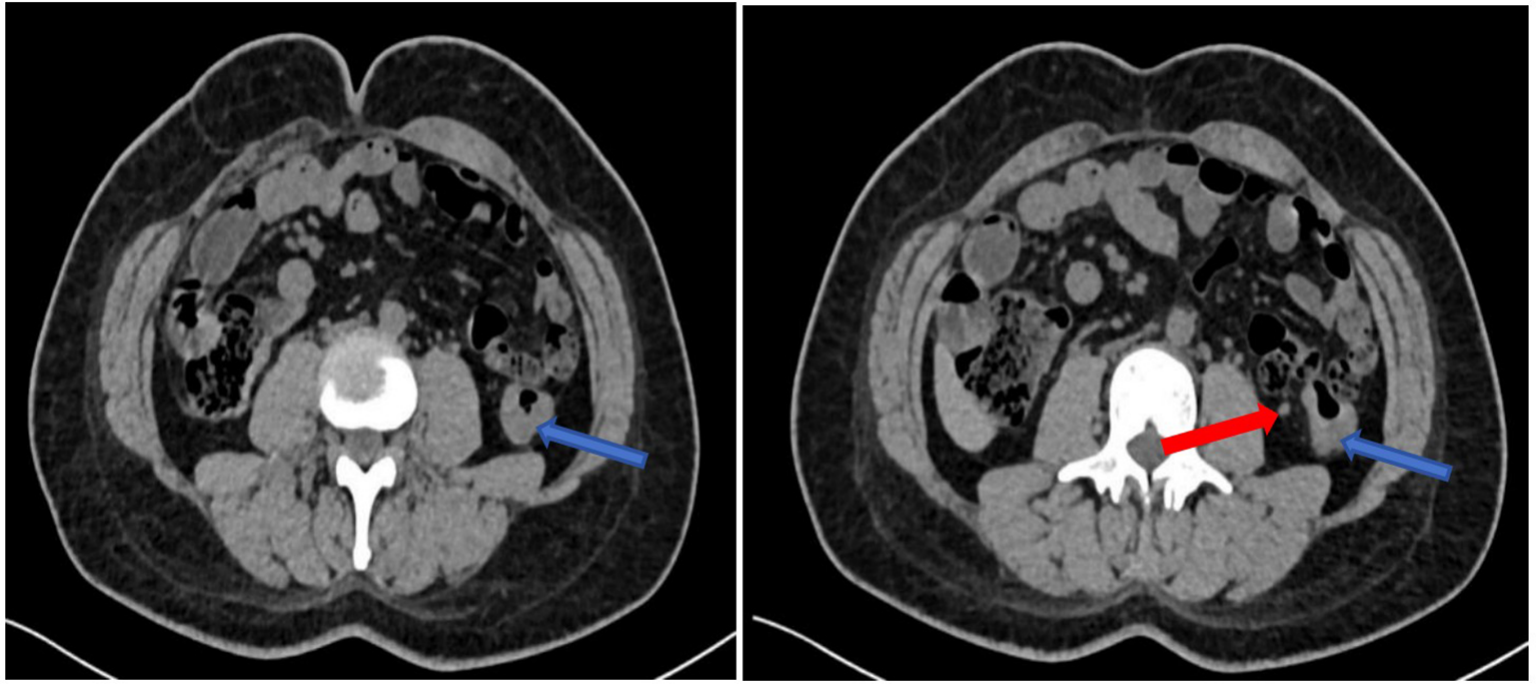
Images from the first CT exam on Feb 22, 2024, with red arrow indicating enlarged lymph nodes and blue arrows marking the tumor site.

CT exam on Feb 22, 2024: Thickening and roughening of the middle descending colon wall, with the thickest area measuring approximately 1.2 cm. Multiple lymph nodes were noted around the bowel, with the largest of which measured 0.7 cm×0.5 cm. Diagnosis: Thickening of the descending colon wall and presence of multiple lymph nodes around the bowel. See **Figure 3**.

**Figure 4:**
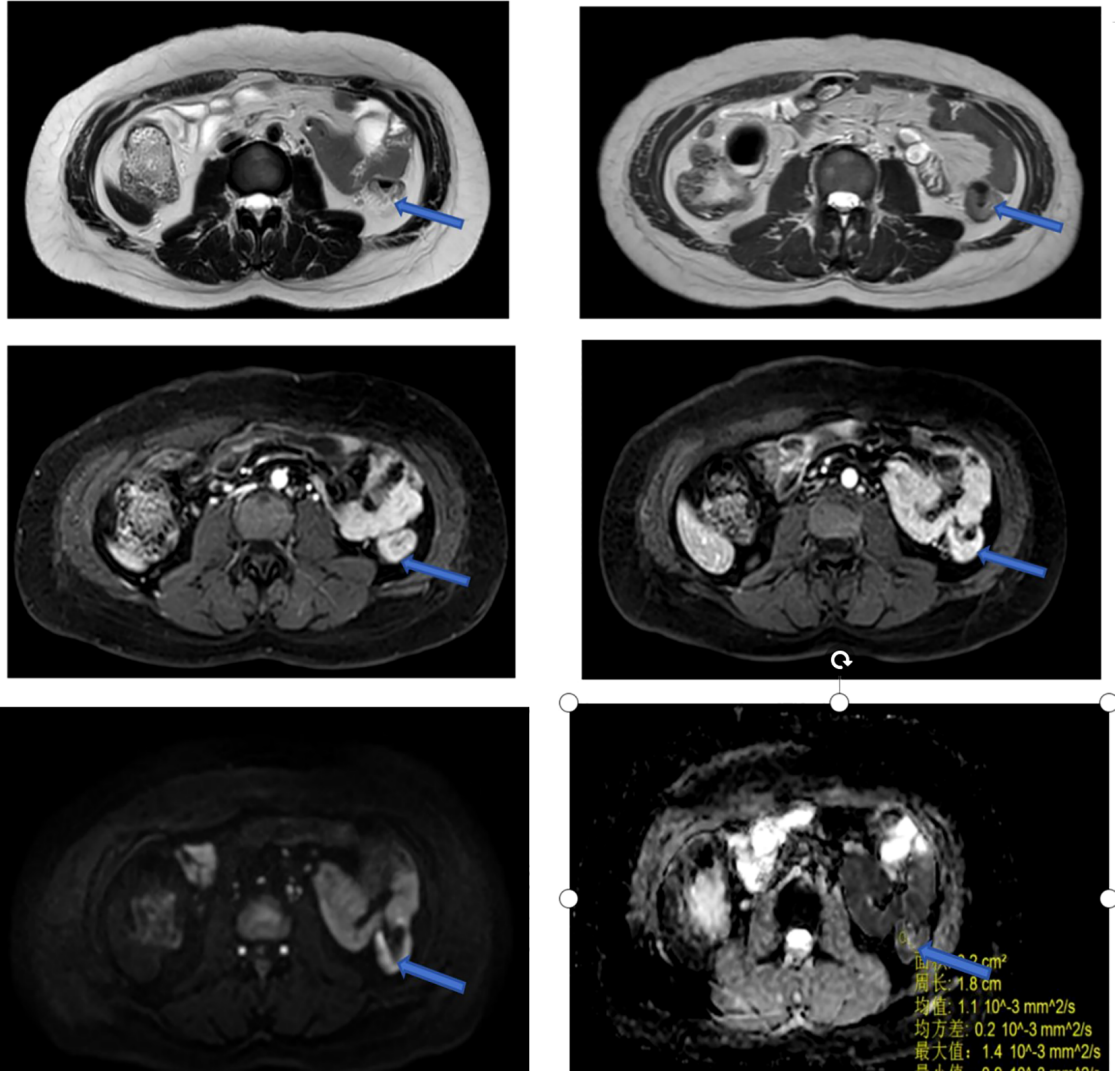
Images from the first MRI exam on Feb 24, 2024, with blue arrows marking the tumor sites.

MRI exam on Feb 25, 2024: Thickening and roughening of the middle descending colon wall, with the thickest area measuring 1.1 cm. Multiple lymph nodes were noted around the bowel, with the largest of which measured 0.3 cm in short diameter. Diagnosis: Mild thickening of the descending colon wall and presence of multiple lymph nodes around the bowel. See **Figure 4**. 

MSI Test report on Feb 28, 2024: The presence of MSI-H. See **Table 1**.

**Table 1:** MSI Test Report.

Test items	Testing method	Tested nucleotide	Nucleotide status	Test result
MSI Testing	PCR + Fragment Analysis	BAT26	Altered	MSI-H
		BAT25	Altered	
		D5S346	Altered	
		D17S250	Unaltered	
		D2S123	Unaltered	

The patient was diagnosed with MSI-H. It is recommended to proceed with further MMR gene-related testing to screen for Lynch syndrome.

Pre-treatment diagnosis: Malignant neoplasm of descending colon (cT4N+M0, Stage III).

CT exam on May 24, 2024: Thickening and roughening of the descending colon wall, with the thickest area measuring approximately 0.9 cm. Adhesion to the left quadratus lumborum muscle was noted. Multiple lymph nodes around the bowel were visible, with the largest being 0.4 cm in diameter.

**Figure 5:**
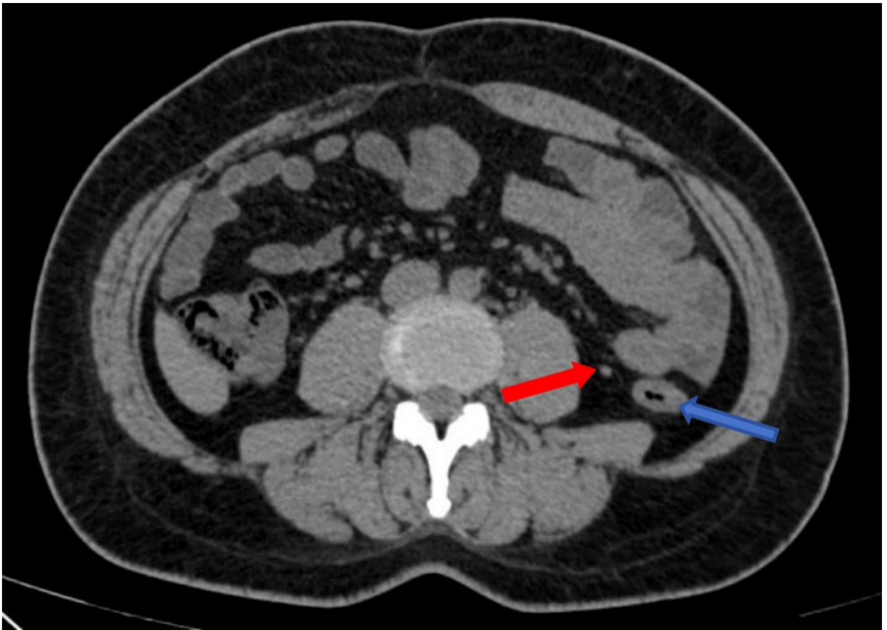
CT exam on May 24, 2024: Red arrow marks enlarged lymph nodes, and blue arrow marks the tumor location.

Diagnosis: “Malignant neoplasm of colon”. Compared to the previous CT scanning image (Feb 22, 2024), the thickening of the descending colon wall had decreased, and the lymph nodes around the colon had reduced in size. See **Figure 5**.

**Figure 6:**
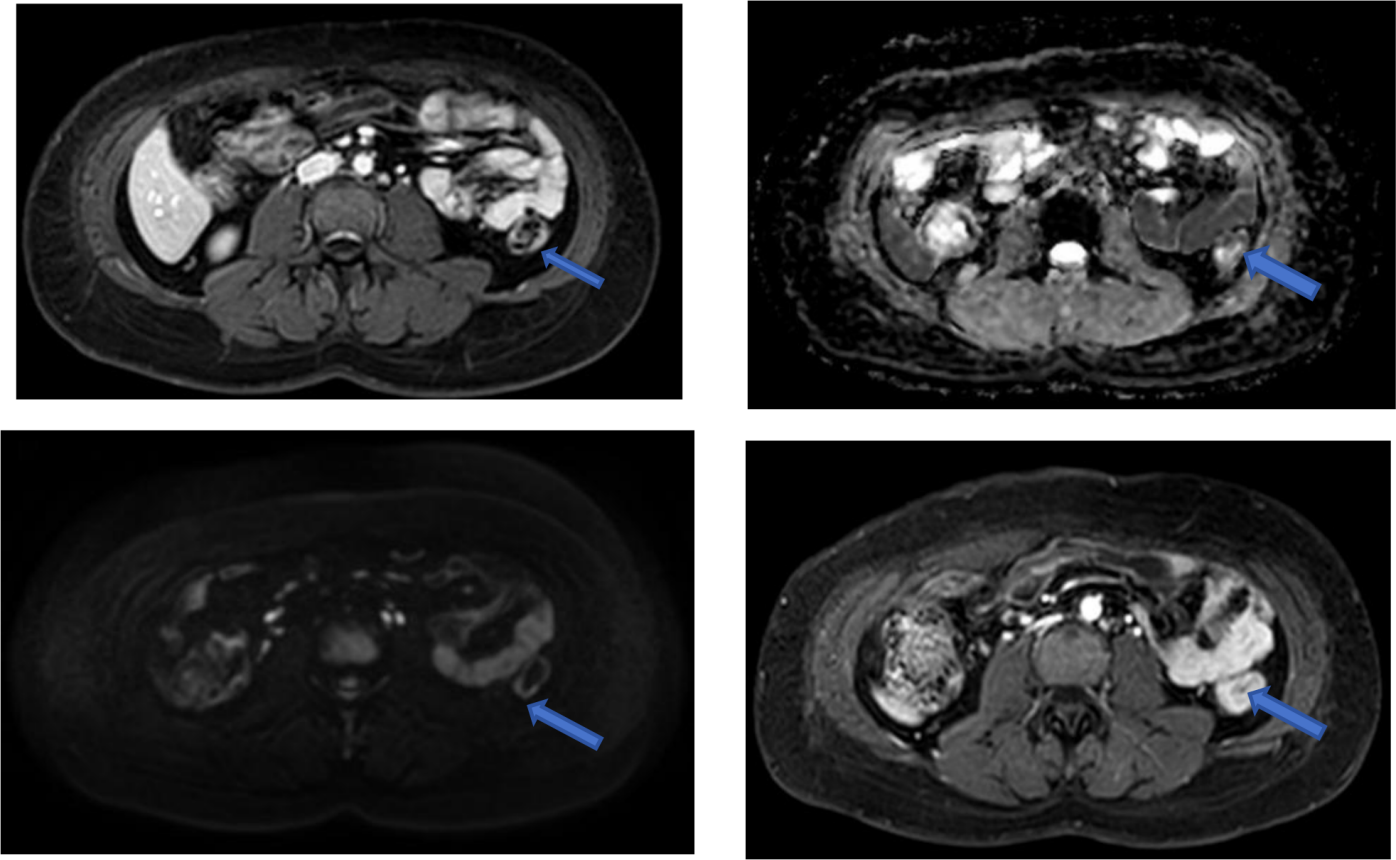
MRI exam on July 4, 2024:Blue arrows mark the tumor locations.

MRI exam on July 04, 2024: Thickening and roughening of the middle descending colon wall, with the thickest area measuring approximately 0.7 cm. Both plain and contrast-enhanced imaging revealed uneven signal intensity. Multiple lymph nodes were noted around the bowel, with the largest measuring approximately 0.3 cm in short diameter. Diagnosis: “Malignant neoplasm of transverse colon”. Compared to the previous MRI on Feb 24, 2024, the descending colon wall showed mild thickening, and the surrounding lymph nodes had decreased in size, and the previously observed lesion had shrunk. See **Figure 6**.

**Figure 7:**
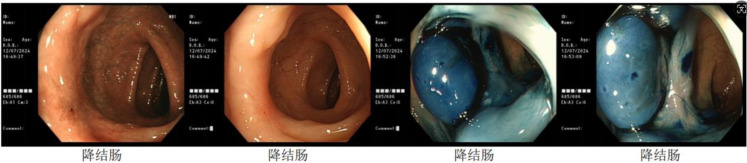
Preoperative colonoscopy on July 4, 2024: Blue-highlighted area indicates the tumor.

Preoperative colonoscopy on July 12, 2024: The anoscope was inserted via the anus, and a scar approximately 2 cm in size was observed in the descending colon about 35 cm from the anal verge. Significant convergence was noted in the surrounding mucosa membrane, and NBI staining showed a loss of the capillary network and glandular openings in the area. See **Figure 7**.

**Figure 8:**
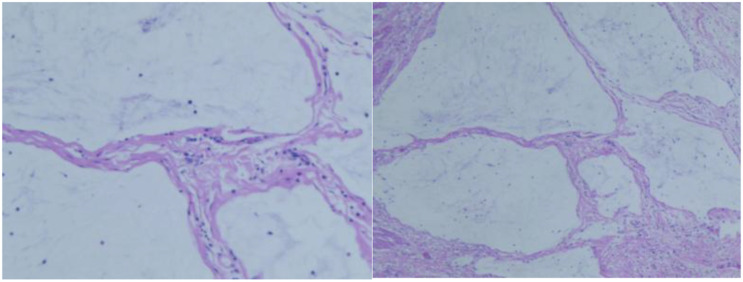
Postoperative pathological examination on July 19, 2024.

At 10:57 AM, July 13, 2024, the patient underwent a laparoscopic left hemicolectomy. Postoperative pathology: <Descending colon & tumor>; ulcerated area: Acute and chronic inflammation with ulceration and abscess formation, and scattered calcium deposits. Mucous lake was formed from the submucosal to subserosa of the bowel wall. No definitive cancerous tissue was identified. Based on the clinical history, these changes were considered as post-treatment changes. No cancer metastasis was found in the lymph nodes sampled from the surrounding fatty tissue (0/3). No cancerous tissue was seen at the two incisal margins of the colon on microscopic examination. No cancerous tissue was observed in the mesenteric lymph nodes (0/11). See **Figure 8**.

## 5 Treatment course & follow-up

Based on the results of imaging, pathological, and genetic tests, the patient was diagnosed with the malignant neoplasm of ascending colon (cT4N+M0), with invasion of the serous membrane of the bowel and the presence of multiple lymph nodes, but no distant metastasis. Given the data from nIT for CRC, nIT could be considered for this MSI-H patient. Based on the discussion of MDT and the latest adjuvant immunotherapy data, it was recommended that the patient received neoadjuvant therapy with ICIs alone. The patient had received Cadonilimab (administered intravenously at a dosage of 6 mg/kg,15-day interval, 9 doses in total) from March 7, 2024, to June 20, 2024. No adverse reactions were noted.

The patient successfully completed nine cycles of neoadjuvant therapy with cadonilimab without experiencing any grade ≥3 treatment-related adverse events. Mild grade 1–2 fatigue and rash were observed, which resolved spontaneously without intervention.

Postoperative pathological evaluation revealed fibrous tissue hyperplasia and chronic inflammatory cell infiltration in the original tumor area, with no viable tumor cells identified (**Figure 8**). No metastatic involvement was found in regional lymph nodes (0/15). According to the “Guidelines for Pathological Assessment of Colorectal Cancer After Neoadjuvant Therapy,” the case was confirmed as a pathological complete response (pCR).

As of the last follow-up in February 2025, the patient remained in a disease-free state with a disease-free survival (DFS) of over 5 months. The ECOG performance status score was maintained at 0, and activities of daily living were fully normal.

Patient-reported outcomes indicated high satisfaction with avoiding traditional chemotherapy and achieving curative effects through short-course therapy. The patient reported that quality of life had returned to pre-illness levels and expressed gratitude to the medical team.

## 6 Discussion

With the advent of precision medicine era, molecular genotyping and microsatellite status in CRC have gained widespread attention and research interest. The microsatellite status of a tumor can be classified as instability (MSS), low instability (MSI-L), or high instability (MSI-H). This variation is typically caused by mutations or loss of expression in key genes (such as MLH1, MSH2, MSH6, and PMS2) within the MMR system. Tumors with dMMR often exhibit MSI-H, meaning that a large number of mutated MSI regions in the tumor. This condition is commonly found in several solid tumors, including endometrial, colorectal, and gastric carcinomas (12). In recent years, ICIs targeting PD-1, PD-L1, or CTLA-4 have emerged as effective treatments for various cancers. Most studies suggest that patients with MSI-H/dMMR solid tumors are more likely to benefit from ICIs. However, the critical factor determining the efficacy of ICI treatment is the MSI-H status rather than the specific cancer type. As a result, both domestic and international guidelines recommend determining a patient’s MSI status before initiating immunotherapy (13, 14).

LACRC carries a high risk of local recurrence and metastasis after surgery. Conventional surgical resection does not meet the clinical treatment needs of these patients. Furthermore, postoperative adjuvant chemotherapy is often limited by postoperative complications and poor physical condition. Consequently, fewer than half of these patients receive adjuvant chemotherapy. To reduce the risk of recurrence and metastasis in LACRC and achieve more thorough resections, the nCT has been proposed as a treatment option for CRC. The FOxTROT trial was the first Phase III study assessing the efficacy of nCT for the resectable colon cancer, showing a significant improvement in the 2-year disease recurrence rate for patients in the nCT group compared to those undergoing direct surgery. However, subgroup analysis indicated that the efficacy of nCT was only 4.7% for MSI-H/dMMR patients, with 73.6% of patients showing no tumor shrinkage; for MSS/pMMR patients, there was only 26.6% patients showing no tumor shrinkage (15). Furthermore, colon cancer patients with dMMR did not benefit from perioperative chemotherapy in terms of disease-free survival (DFS) when compared to patients with pMMR. This suggests that MSI-H/dMMR LACRC has distinct clinical, pathological, and molecular characteristics. These patients are less likely to benefit from nCT compared to patients with pMMR. However, the KEYNOTE-177 study on the exploration of first-line treatment for advanced CRC (16) demonstrated that ICIs had a clear advantage over conventional chemotherapy combined with targeted therapy in MSI-H/dMMR tumors. This suggested that the use of ICIs in neoadjuvant therapy for LACRC could lead to more promising treatment outcomes. In recent years, multiple studies on the nIT for MSI-H/dMMR CRC have been conducted both domestically and internationally, with all reporting exciting results with high rates of cCR and pCR.

The NICHE trial (17) was the first study on the nIT for resectable colon cancer, enrolling 30 subjects with pMMR colonic cancers, and 32 with dMMR colonic cancers for receiving Nivolumab and Ipilimumab for 6 weeks. Among them, patients with pMMR were randomly assigned to receive Celecoxib. In the 32 patients with dMMR, 78% were N+, and the clinical T staging distribution was as follows: 19% of T2, 31% of T3, and 47% of T4, indicating that only 47% of patients had T4 stage disease. All dMMR patients showed pathological responses, with a major pathological response (MPR) rate of 97% (31/32) and a pCR rate of 69% (22/31). After a median follow-up of 32 months, no patients in the dMMR cohort experienced disease recurrence. Among all patients, 12% experienced grade 3 immune-related adverse events (irAEs) and no grade 4 irAEs or surgical complications were reported. Based on the favorable signals observed in a small group of patients with dMMR colon cancers in the NICHE trial, the Dutch investigator Myriam Chalabi et al. (18) conducted a large Phase II NICHE-2 study to validate the safety and efficacy of neoadjuvant treatment with Nivolumab and Ipilimumab for 6 weeks in the subjects with previously untreated, non-metastatic dMMR LACC. The primary endpoints of the study were the safety and the 3-year DFS rate. At baseline, 74% of patients were staged as high-risk Stage III, with 35% having T4a tumors and 28% having T4b tumors. More than half of the patients (62%) had N2 stage. Of the 115 subjects enrolled, 4% experienced grades 3–4 irAEs, and 2 patients had surgery delayed by more than 2 weeks. Among the 111 subject who were included in the efficacy analysis, 98% showed pathological responses, with a MPR rate of 95% and a pCR rate of 68%. After a median follow-up of 26 months, no patients had a recurrence of disease. The NICHE-2 trial demonstrated a pCR rate similar with that observed in the NICHE study. In contrast, the Phase II PICC trial led by Hu Huabin et al. at the Sixth Affiliated Hospital of Sun Yat-sen University (19) was aimed to explore the efficacy and safety of neoadjuvant monotherapy with ICIs in MSI-H/dMMR LACRC. The study enrolled 34 MSI-H/dMMR CRC subjects with clinical staging of T3-T4 or any T stage with lymph node metastasis positive (26% T3 and 74% T4). These patients received neoadjuvant therapy with Toripalimab alone or combined with Celecoxib (a COX-2 inhibitor) for 6 cycles. The results showed that the pCR rate in the combination therapy group (Nivolumab plus Ipilimumab) was 88%, compared to 65% in the Toripalimab monotherapy group. The overall pCR rate was 76.5%, with a favorable safety profile. Among the 34 patients, only one experienced a grade 3 adverse event (AST increased). When compared to the NICHE and NICHE-2 studies, the PICC study demonstrated a pathological response rate for the neoadjuvant immune monotherapy (3 months) similar to the neoadjuvant dual-ICI therapy (6 weeks). However, the preoperative duration was doubled. In terms of safety, the immune monotherapy showed better safety control, with fewer grade 3 irAEs.

Based on the results from the PICC and NICHE-2 studies, the CSCO Guidelines for Diagnosis and Treatment of Colorectal Cancer (2023) recommends the use of ICIs (PD-1 ± CTLA-4) for dMMR/MSI-H patients at cT4b, followed by radical surgery. Previous studies have shown that neoadjuvant therapy with ICIs for dMMR/MSI-H LACRC significantly improves outcomes compared to conventional chemoradiotherapy. ICI therapy not only leads to a higher pCR rate but also reduces the incidence of adverse events, thereby improving organ function. Considering that this patient had high-risk T4b stage of descending colon cancer with late clinical staging and dMMR status confirmed by immunohistochemistry, the Cadonilimab targeting both PD-1 and CTLA-4 was selected for neoadjuvant therapy, with the hope that the bispecific antibody would offer better therapeutic outcomes for T4 colon cancers. Although T4 colon cancer (tumor penetrating the colonic serosa) accounts for only 10%-15% of cases, the prognosis for T4 colon cancer remain poor (20, 21). The 3-year recurrence rate for T4 colon cancer remains at 47%, whereas it is only 13% for T1-T2 stage colon cancer (22). Furthermore, Dr. Andrea Cercek has highlighted that staging of dMMR colon cancer is particularly challenging, as tumors with MSI often exhibit lymph node enlargement due to immune infiltration, leading to potential false positives (7). Neoadjuvant therapy has shown promise in improving outcomes for T4 colon cancer, a strategy that has proven effective in gastric and rectal cancers, where it helps reduce tumor cell shedding during the surgical resection process (5). In this case, after 9 cycles of Cadonilimab, the patient achieved pCR, marking the first time Cadonilimab has been used for neoadjuvant therapy in T4 colon cancer, resulting in pCR without any adverse effects. Although the incidence of grade 3 or 4 adverse events in the NICHE-2 study with dual-ICI therapy was 4%, 11% of patients experienced long-term endocrine dysfunction (5, 18). Cadonilimab demonstrated noteworthy efficacy and safety profiles in this case report. Compared with key clinical trial data from existing neoadjuvant immunotherapy studies for dMMR/MSI-H colorectal cancer, its performance exhibits distinct characteristics. Regarding efficacy, the patient achieved a pathological complete response (pCR) following neoadjuvant therapy with cadonilimab. This outcome aligns with the trend of high pCR rates reported in major clinical studies, though with some variations. Specifically, the NICHE-2 trial (nivolumab plus ipilimumab) reported a pCR rate of 68% in patients with dMMR colon cancer (23), while the PICC trial (sintilimab monotherapy) reported a pCR rate of 88.9% (16/18) in its dMMR/MSI-H colorectal cancer cohort (6). The result from this single case preliminarily supports the potential of cadonilimab, as a PD-1/CTLA-4 bispecific antibody, to induce deep tumor remission. In terms of safety, the absence of any grade ≥3 treatment-related adverse events in this case is particularly remarkable. In comparison, the incidence of grade ≥3 treatment-related adverse events was 25% in the NICHE-2 study employing combination immunotherapy (nivolumab + ipilimumab) (1). The PICC trial with sintilimab monotherapy reported a lower incidence of grade ≥3 toxicity, approximately 5.6% (1/18) (6). The favorable safety profile observed in this case aligns with the design rationale of cadonilimab, which aims to reduce peripheral toxicity mediated by CTLA-4 targeting through its unique structure (lacking Fc effector function and enriched in the tumor microenvironment). This preliminary finding suggests that cadonilimab may represent a novel neoadjuvant treatment option combining robust efficacy with an improved safety profile. Cadonilimab, as a novel bispecific antibody, eliminates Fc receptor- and complement-mediated cytotoxic effects. With its higher affinity for lymphocytes in the tumor micro-environment compared to peripheral sites, it can inhibit CTLA-4 binding in peripheral blood and normal tissues, reducing the toxic side effects caused by the CTLA-4 inhibitors. Compared to PD-1 and CTLA-4 combination therapy, Cadonilimab significantly reduces toxicity.

## 7 Conclusion

This is the first reported dMMR/MSI-H CRC case who has been treated with neoadjuvant Cadonilimab monotherapy and achieved pCR. The treatment is well tolerated, and the safety profile is manageable. Future studies with larger sample sizes and a greater number of participants are needed to further explore the therapeutic role of Cadonilimab in dMMR/MSI-H CRC.

## Data Availability

The original contributions presented in the study are included in the article/supplementary material, further inquiries can be directed to the corresponding author/s.
