# Correction: Neoadjuvant therapy with cadonilimab in a patient with MSI-H/dMMR colorectal cancer: a case report

**DOI:** 10.3389/fonc.2026.1844247

**Published:** 2026-04-13

**Authors:** Jingrui Zhou, Weimin Chen, Jue Wang, Ziwei Chen, Guanghui Guo, Tao Yang, Tao Shen

**Affiliations:** Department of Colorectal Surgery, Yunnan Cancer Hospital, The Third Affiliated Hospital of Kunming Medical University, Peking University Cancer Yunnan Hospital, Kunming, Yunnan, China

**Keywords:** colorectal cancer, cadonilimab, immune checkpoint inhibitors, neoadjuvant immunotherapy, pathological complete response, programmed death-1 (PD-1), cytotoxic T-lymphocyte-associated antigen 4 (CTLA-4)

An incorrect number was provided for Yunnan Province Science and Technology Department - Youth Academic and Technical Leader Reserve Talent Program. The correct number is 202405AC350003.

The original version of this article has been updated.

